# Genome-wide search for Zelda-like chromatin signatures identifies GAF as a pioneer factor in early fly development

**DOI:** 10.1186/s13072-017-0141-5

**Published:** 2017-07-04

**Authors:** Arbel Moshe, Tommy Kaplan

**Affiliations:** School of Computer Science and Engineering, The Hebrew University of Jerusalem, Jerusalem, 91904 Israel

**Keywords:** Zelda, Maternal-to-zygotic transition, Histone modifications, Enhancers, Chromatin, Spectral clustering, Chromatin search, GAGA factor

## Abstract

**Background:**

The protein Zelda was shown to play a key role in early Drosophila development, binding thousands of promoters and enhancers prior to maternal-to-zygotic transition (MZT), and marking them for transcriptional activation. Recently, we showed that Zelda acts through specific chromatin patterns of histone modifications to mark developmental enhancers and active promoters. Intriguingly, some Zelda sites still maintain these chromatin patterns in Drosophila embryos lacking maternal Zelda protein. This suggests that additional Zelda-like pioneer factors may act in early fly embryos.

**Results:**

We developed a computational method to analyze and refine the chromatin landscape surrounding early Zelda peaks, using a multichannel spectral clustering. This allowed us to characterize their chromatin patterns through MZT (mitotic cycles 8–14). Specifically, we focused on H3K4me1, H3K4me3, H3K18ac, H3K27ac, and H3K27me3 and identified three different classes of chromatin signatures, matching “promoters,” “enhancers” and “transiently bound” Zelda peaks. We then further scanned the genome using these chromatin patterns and identified additional loci—with no Zelda binding—that show similar chromatin patterns, resulting with hundreds of Zelda-independent putative enhancers. These regions were found to be enriched with GAGA factor (GAF, Trl) and are typically located near early developmental zygotic genes. Overall our analysis suggests that GAF, together with Zelda, plays an important role in activating the zygotic genome.

**Conclusions:**

As we show, our computational approach offers an efficient algorithm for characterizing chromatin signatures around some loci of interest and allows a genome-wide identification of additional loci with similar chromatin patterns.

**Electronic supplementary material:**

The online version of this article (doi:10.1186/s13072-017-0141-5) contains supplementary material, which is available to authorized users.

## Background

The process of transcription is vital to all living organisms and is tightly regulated by multiple mechanisms, including the packaging of DNA into chromatin. In eukaryotic cells, the DNA is wrapped around nucleosomes to form chromatin. This packaging is used differentially to control in what conditions and cell types a gene is more accessible—and active—and in which conditions it is tightly packed and silenced. This packaging of DNA was shown to be mediated by various mechanisms, including the deposition of covalent modifications (e.g., acetylation, methylation, phosphorylation or ubiquitylation) at different residues of the core histone proteins that are assembled into a nucleosome [1]. These histone modifications influence various processes along the DNA. For example, H3K4me1 and H3K27ac (namely, mono-methylation of Lysine 4 or acetylation of Lysine 27 in histone H3) are found at nucleosomes carrying active regulatory regions, while H3K27me3 is known to be a repressive mark [2–4].

The packaging of DNA into chromatin, including nucleosome positioning and their histone modifications, are fundamental to the proper activity of regulatory regions, by controlling which regions of the genome are accessible for protein binding and which are not [5–7]. Of specific interest are promoter regions, located near the transcription start site (TSS) of genes; and enhancers, that regulate gene expression from afar, often up to 1 Mb away from their target genes. In addition, chromatin marks and structural proteins allow distal enhancers to fold in 3D into close spatial proximity to their target genes [8–10].

Comparison of tissue- and condition-specific chromatin data highlights the tight regulation of gene expression by chromatin, determining which genomic regions are active and which are not, and as a consequence which genes will be transcribed. This raises the question of causality. Who regulates packaging? Or how does the genome get packed initially in the proper architecture, e.g., to drive early developmental expression?

In practically all animals, early developmental stages begin with maternal proteins and RNA that control the first hours in the fertilized egg. At this stage, these proteins control the first wave of zygotic expression and direct the first mitotic divisions [11]. After that, the embryo undergoes a process called maternal-to-zygotic transition (MZT), in which the zygotic genome is activated and takes control of mRNA and protein production. Finally, maternal mRNA and proteins are degraded.

In the fruit fly *Drosophila melanogaster*, embryonic development is characterized by a series of 13 rapid replication cycles, occurring during the first 2 h after fertilization. The division of cells slows at the 14th mitotic cycle, and zygotic transcription initiates. This marks the end of the *D. melanogaster* maternal-to-zygotic transition. This process is crucial for the normal development of the embryo and is tightly controlled, in both time and space, by the gradual activation of a cascade of transcription factors [12, 13]. This required multiple molecular mechanisms that include chromatin, nucleosomes, DNA accessibility, steric hindrance between DNA-binding proteins and more.

**Fig. 1:**
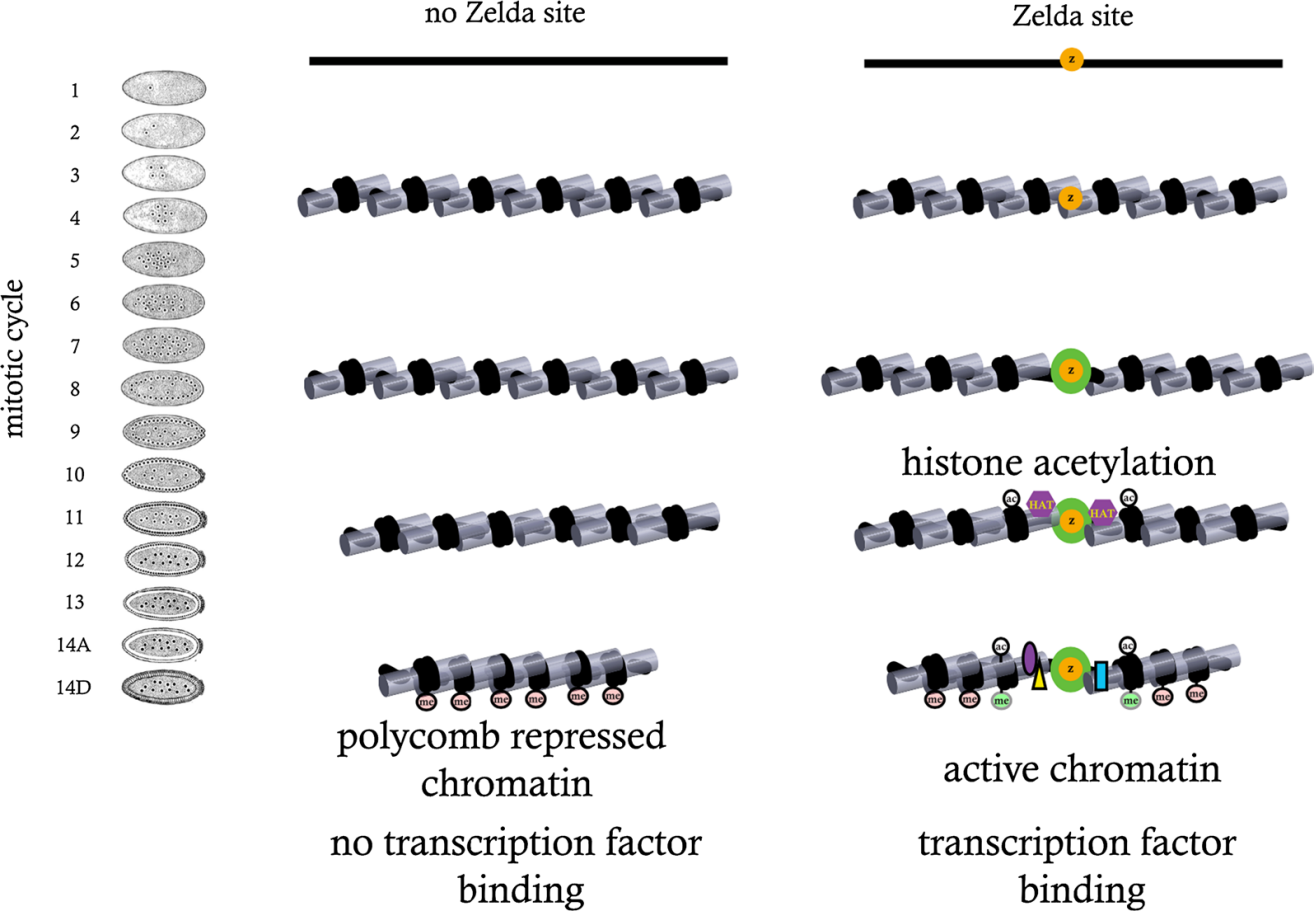
The role of Zelda during zygotic genome activation. Zelda binds to regulatory regions in pre-MZT embryos as early as mitotic cycle 2. This leads to histone acetylation and nucleosome remodeling around ZLD binding sites, which facilitates binding by other transcription factors and deposition of active histone marks. At the same time, Zelda binding prevents deposition of H3K27me3 marks and formation of repressive chromatin structure. Adapted from Li et al. [5]

Previous research showed that many of the early transcribed genes in Drosophila embryos contain a specific DNA motif of 7 bp, CAGGTAG, that occurs within their regulatory regions, including both promoters and enhancers [14–16]. This motif was later identified to be the binding site of the zinc-finger transcription factor Zelda (vielfaltig, vfl) [17]. Following studies, by us and others, showed that Zelda is present in the embryonic nucleus as early as mitotic cycle 2 and binds thousands of genomic loci, including the promoters and enhancers of thousands of early developmental genes (Fig. 1) [12, 16–18].

Computational and experimental studies, by us and others, suggested that Zelda acts as a pioneer factor, binding mostly inaccessible DNA regions and making the chromatin accessible for other transcription factors to bind, thus marking thousands of genes and regulatory regions for activation which drives the first transcriptional program of the developing embryo [9, 16, 19].

Indeed, experimental studies showed that early Zelda binding is a predictor for open chromatin and transcription factor binding at mitotic cycle 14 [13, 15–17, 20]. The entire set of molecular mechanisms by which Zelda functions to access the genome and mark it for activation is yet to be discovered, as is the role of additional proteins in this crucial stage in embryonic gene expression.

Several studies mapped the chromatin landscape in *D. melanogaster*, most of which in cells or during very broad temporal windows [21–24]. Of particular interest are few studies, in which early and manually staged Drosophila embryos were used to portray Zelda binding locations and multiple histone marks (including H3K27ac, H3K4me1, H3K4me3, H327me3, and H3K36me3) at several time points throughout MZT and early embryonic development [5, 16]. As was shown, early Zelda binding often results in open chromatin and characteristic histone marks, including H3K27ac, H3K4me1, and H3K27me3 peaks [17, 25].

Intriguingly, the causal role of Zelda in proper establishment of chromatin domains and normal gene expression was also examined. Embryos lacking zygotic Zelda expression as well as embryos with mutations in specific Zelda binding sites (near early genes) showed developmental abnormalities [14, 17]. Embryos lacking maternal Zelda expression (zld^M−^) showed reduced accessibility for many, but not all, distal enhancers regulating early fly development, while maintaining near-normal promoter accessibility [5, 20].

The latter results suggest that perhaps, in addition to Zelda, there might be another protein that marks early developmental genes for activation. To answer this question, we revisited the chromatin patterns surrounding Zelda binding sites and developed a computational statistical model to characterize histone modifications and their dynamics. Several previous methods faced a similar situation, where a set of genomic loci are given, and unsupervised machine learning techniques should be used to re-orient them [26] (e.g., for asymmetric chromatin signatures, often found at transcription start sites), re-align them using profile alignment dynamic programming algorithms [27], and cluster them into several distinct classes. At a second stage, we used our computational model to scan the genome and identify additional genomic loci that show similar chromatin patterns. Previous algorithms (e.g., RFECS [28]) used machine learning to train a set of binary classification trees (random forest) and classify the chromatin data around each putative enhancer locus (±1 kb window). Instead, we applied a regularized correlation-based approach, where each genomic locus is compared to the (multidimensional) chromatin signature of each cluster. This allowed us to maintain the overall typical shape of each histone modification, while allowing for different ChIP signal intensities.

As we show, our approach identified about 2000 genomic regions with Zelda-like chromatin signatures and dynamics, with no Zelda binding. As we show, these regions are enriched for the protein GAF, whose early binding could establish open chromatin and activation of regulatory regions.

## Results

To identify additional Zelda-like regulators that act as pioneer factors during early Drosophila development, we begin by characterizing the chromatin landscape induced around early Zelda sites.

### Zelda peaks vary in their chromatin signatures

We have previously shown that early Zelda peaks, identified via ZLD ChIP-seq in hand-sorted fly embryos from mitotic cycle 8, are associated with open chromatin regions and transcription factors binding later on, toward the end of the maternal-to-zygotic transition (mitotic cycle 14) (Fig. 1) [12, 13, 16].

**Fig. 2:**
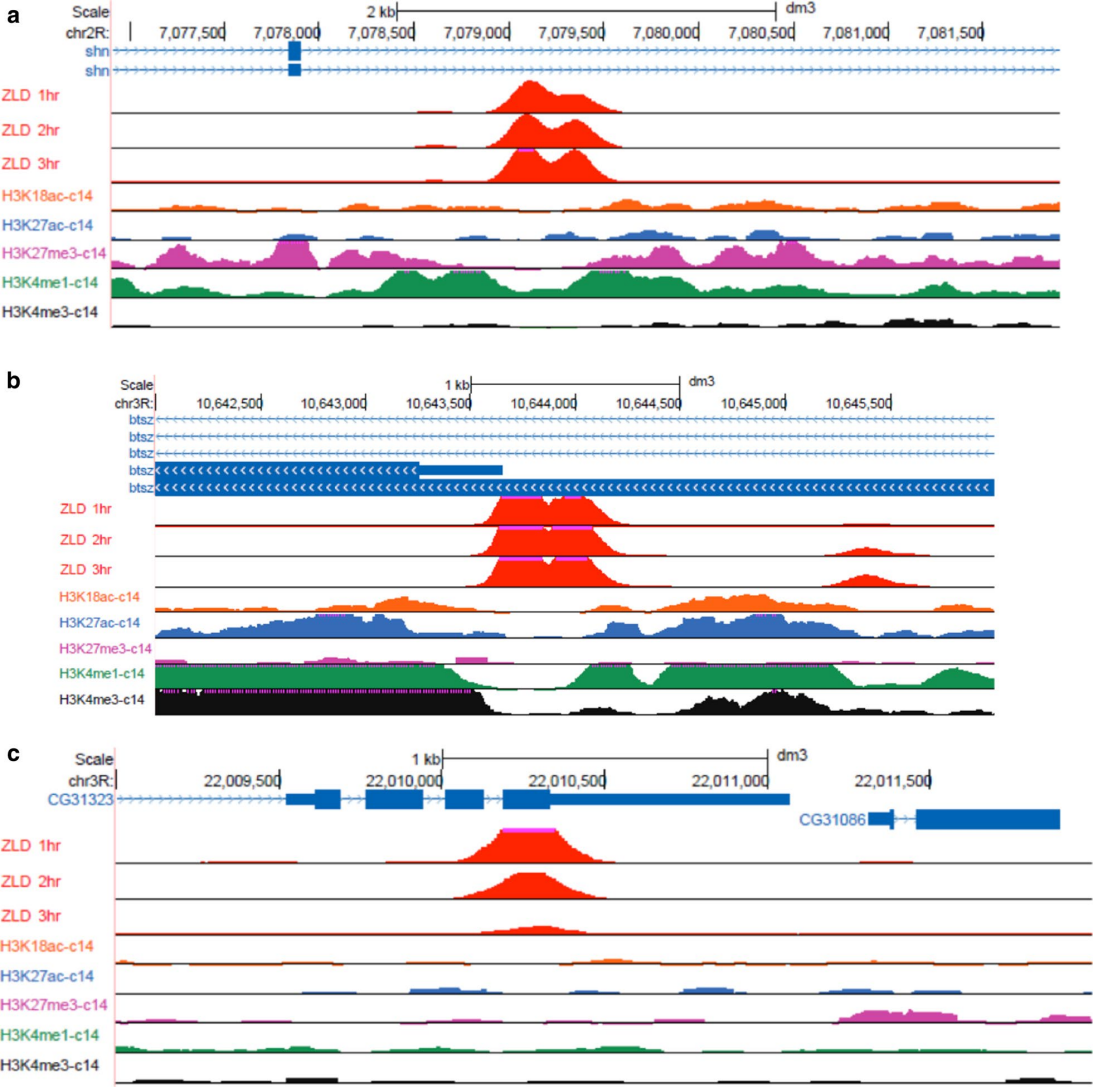
Early Zelda peaks show different chromatin signatures. Shown are three examples of ZLD peaks, each with a different chromatin signature. **a** Shown is a distal enhancer of the *schnurri* gene (*shn*), where Zelda binds an intronic enhancer (also bound by multiple anterior–posterior transcription factors, not shown), is flanked by H3K4me1 nucleosomes (*green*) within an H3K27me3 domain (*purple*). **b** Shown is the promoter of the *bitesize* gene (*btsz*), highlighted with the typical promoter modification (H3K4me3, *black*) and an asymmetric H3K4me1 domain (*green*), with flanking H3K27ac and H3K18ac nucleosomes (*blue*, *orange*). **c** Shown is a ZLD peak overlapping the coding region of an inactive gene, with zygotic expression smaller than 0.2 FPKM in mitotic cycles 10–14 [30]. Zelda seems to be transiently bound, with very weak binding by the end of mitotic cycle 14 [16]. No histone modifications are observed along the  neighboring regions

However, a close examination of some strong early Zelda sites (Fig. 2) suggests that there is more than one typical chromatin signature. Specifically, shown are two Zelda peaks, one at an intron of the *schnurri* gene (*shn*, Fig. 2a) and one at the promoter of *bitesize* (btsz, Fig. 2b). The former shows a ZLD peak surrounded by H3K4me1-marked nucleosomes and flanked by H3K27 tri-methylation. The latter shows the promoter Zelda peak is surrounded by H3K4me1 nucleosomes, as well as H3K4me3 on one side of the ZLD peak. Intriguingly, as shown in Fig. 2c, some early Zelda peaks seem to show no chromatin pattern whatsoever (by cycle 14) and are practically indistinguishable from their surroundings.

We therefore hypothesized that different Zelda peaks could have different histone modification patterns, which may not be obscured when only considering the average, common pattern, near Zelda peaks.

### Chromatin-based re-orientation of early Zelda peaks

As a first step toward a more descriptive and accurate characterization of chromatin near Zelda sites, we first wanted to check which histone modifications are symmetric around Zelda peaks and which are not [26]. For this, we implemented an expectation maximization (EM)-like iterative orientation method that automatically infers, based on histone modification data, the orientation of each Zelda peak, while iteratively improving the typical chromatin “signature” surrounding Zelda peaks [26, 29].

**Fig. 3:**
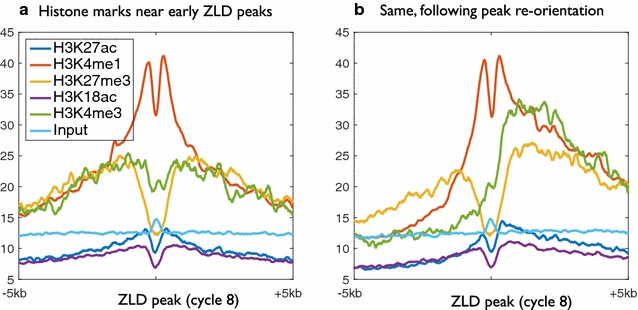
Chromatin marks surrounding Zelda peaks prior to and following peak re-orientation. **a** Average chromatin marks at mitotic cycle 14, from Li et al. [5], centered on 2000 early Zelda peaks (cycle 8). Clearly visible are relative enrichment near Zelda site for the enhancer mark H3K4me1 (*orange*) and depletion of the repressive H3K27me3 (*yellow*). **b** Same, following re-orientation of Zelda peaks based on all five modifications

Our algorithm begins with a random orientation of each Zelda site. We then calculate the average ChIP-seq signal over all Zelda peaks in a 10 Kb region (Fig. 3a). Then, every iteration, we enumerate over each Zelda peak and compare the similarity of its surrounding 10 Kb for the average signal in each of the two orientations (+ or −). This is done while considering all chromatin marks together. Regions with higher similarity for the inverted signatures are then flipped. Finally, we calculate the updated average chromatin signatures, and a new iteration begins. Figure 3b shows the final chromatin signatures, after the algorithm converges (typically, in less than 10 iterations).

We decided to choose a fully unsupervised chromatin-based re-orientation, rather than relying on gene directionality, as we believe that at least some of the modifications are “one-sided,” and therefore would contribute to inferring the orientation of most Zelda peaks. Indeed, while most of the modifications maintain a symmetrical formation, both H3K4me3 (promoter) and H3K36me3 (gene body) show strong asymmetric bias, consistent with the orientation of the underlying gene (Fig. 3b). Also notable is the input IP signature, which is almost entirely flat (except for a tiny bump near Zelda), suggesting that the asymmetric signatures are not due to the additional degree of freedom per Zelda site.

### Chromatin-based clustering of Zelda peaks

To test whether there are different types of Zelda peaks, each with a different combination of histone modifications patterns, we turned to develop a computational model that will allow us to characterize the chromatin landscape and temporal dynamics at each early Zelda peak. Such an approach would not only shed light on the different functional roles of Zelda peaks, but would also allow us to scan the genome using a refined model, and identify additional loci, independent of ZLD binding, that undergo similar chromatin dynamics during MZT.

As shown in Figs. 2 and 3, it is not enough to only consider the maximal signal of each histone modification, as the modifications we look at are not characterized by a single peak, but rather show a unique spatial signature, which is often asymmetric. We therefore want a specialized method that considers both the pattern of each histone modification and the overall combination of different modifications and their heights.

For this, we decided to examine a 10-Kb window surrounding each Zelda peak and apply a spectral clustering algorithm that will consider all histone modifications simultaneously. We begin by describing a distance function between the chromatin signature of two loci that would capture both the “landscape shape” of each modification, its overall magnitude, and the combinatorial nature of multiple modifications.

An advantage of spectral clustering is the fact that the algorithm relies on the adjacency (or connectivity) between objects within each cluster, rather than their spatial shape.

Our initial chromatin ChIP-seq data consisted of nine histone modifications (H3K4ac, H3K4me1, H3K4me3, H3K9ac, H3K18ac, H3K27ac, H3K27me3, H3K36me3, and H4K5ac) in four time points throughput MZT (mitotic cycles 8, 11, 13, and 14) [5]. We therefore selected the top 2000 early (cycle 8) Zelda peaks—prior to MZT and the activation of most zygotic genes, and before the establishment of chromatin marks [5, 30]—and for each considered a surrounding window of 10 Kb for five regulatory histone modifications (H3K4me1, H3K4me3, H3K27ac, H3K27me3, and H3K18ac), as measured in mitotic cycles 13 and 14. H3K36me3 was often depleted from Zelda sites and localized mainly in gene bodies, and we decided not to use it for the clustering. The idea here was not to obtain a definitive metric for chromatin, but rather to identify the regulation-specific chromatin landscapes near Zelda peaks.

**Fig. 4:**
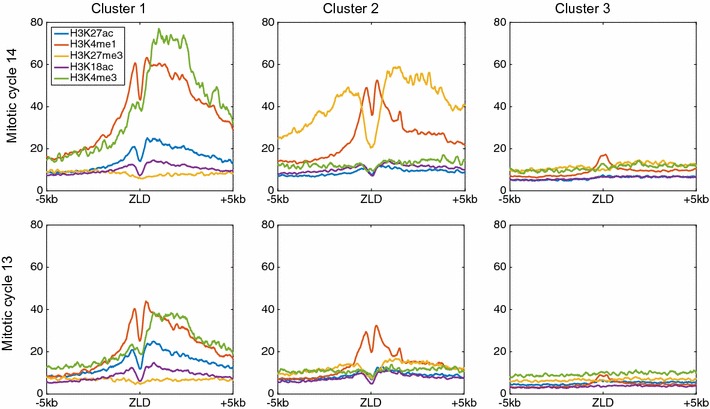
Spectral clustering of chromatin signatures for top 2000 early Zelda sites. Cluster 1 (*left*) is composed of 620 early Zelda peaks, with an average chromatin signal of asymmetric H3K4me3 with H3K4me1 mark (promoter-like signature). Cluster 2 includes 660 early Zelda peaks and shows symmetric signature of two flanking H3K4me1 nucleosomes, within a larger H3K27me3 region. Cluster 3 (*right*) includes 720 Zelda peaks and is characterized by almost no modifications. *Top row* chromatin data from mitotic cycle 14; *bottom* mitotic cycle 13

This resulted in representing each genomic locus i by ten vectors **x**_**i**_^**m**^: one for each of the five histone modifications at each of the two time points (marked by m). We have down-sampled the chromatin data to 10 bp resolution, resulting in vectors of length 1000. To calculate the distance **dist**_***i,j***_^***m***^ between the two vectors representing the landscape of the modification m surrounding two loci *i* and *j*, we first used the root mean squared deviation (RMSD, see Eq. 1, “Methods”) between the two vectors **x**_**i**_^**m**^ and **x**_**j**_^**m**^ and then converted these into weights (or adjacency scores), using a Gaussian kernel with a modification-specific parameter **σ**_**m**_ (Eq. 2, “Methods”). This parameter sets the standard deviation parameter of the Gaussian kernel, thus normalizing different histone modifications and assigning each a similar importance. We have arbitrarily set **σ**_**m**_ for each modification/time point to equal to the 10th percentile in the distribution of pairwise distances for that specific modification (see “Methods”). Finally, we summed each of the ten adjacency matrices (Eq. 3) and applied standard (symmetrically normalized) spectral clustering, followed by *k*-means [31]. The value for ***k*** (i.e., number of clusters) was chosen using the eigengap heuristic (“Methods”). Overall, the clustering process resulted with three main clusters, each with a unique combinatorial chromatin signature (Fig. 4 and Additional file 1: Figure S1) . Similar results with different bin sizes (20 bp, 50 bp, 100 bp) resulted with almost identical orientation or cluster assignment (<1% change).

The first cluster (Fig. 4, left) is composed of 620 early Zelda peaks (of the initial 2000 peaks analyzed) and shows enrichment for H3K4me1 (orange), on either side of early Zelda peaks, and H3K4me3 mark (green), enriched only downstream of Zelda peaks. While these marks are already observed in mitotic cycle 13 (bottom row), they dramatically strengthen by cycle 14 (top) [5].

The second cluster (Fig. 4, center, 660 Zelda peaks) shows narrow symmetric H3K4me1 peaks (orange) on either sides of Zelda, flanked by wider H3K27me3 domains (yellow). Intriguingly, while this pattern resembles the chromatin signature of “poised enhancers,” described by Rada-Iglesias et al. [3] in human embryonic stem cells (hESCs), a closer examination suggests that most of these genomic regions are actually active enhancer regions (see below). As we noted previously [5], H3K27me3 is deposited almost exclusively by mitotic cycle 14, following MZT.

Finally, the third cluster (Fig. 4, right, 720 Zelda peaks) showed almost no enriched chromatin marks, suggesting that these are transient Zelda binding sites that are bound early and then vacated with no apparent biological effect [5].

### Functional characterization of three Zelda clusters

As we showed, spectral clustering of early Zelda peaks resulted in three clusters, each displaying a unique combination of histone modifications. We next turned to examine whether these clusters demonstrate different functional parameters.

**Fig. 5:**
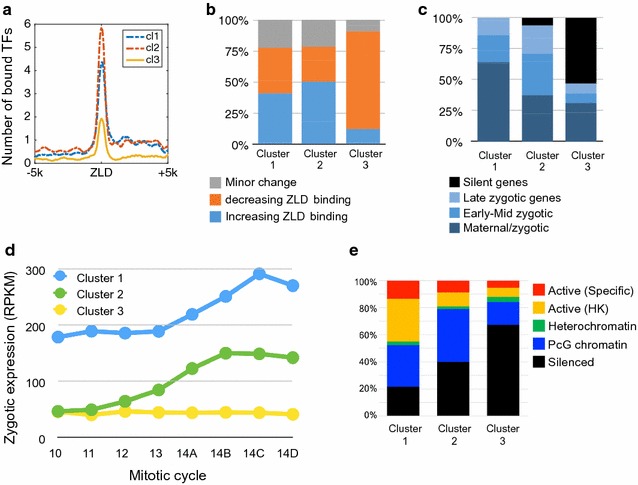
Biological characteristics of the three clusters. **a** By examining the binding of 17 anterior–posterior and dorsal–ventral transcription factor (TFs) in early fly development, we calculated the average number of TFs bound for each locus with each cluster [13]. Early Zelda peaks in clusters 1 and 2 seem to be bound (by the end of mitotic cycle 14) by >4 and ~6 factors, respectively, compared to <2 factors bound near early Zelda peaks in cluster 3. **b** Analysis of temporal dynamics in Zelda binding [16]. In clusters 1 (*left*) and 2 (*center*), about 37% of the ZLD peaks are increasing, 40% decreasing, and ~20% show minor changes in peak height. In contrast, 79% of cluster 3 regions (*right*) show reduced ZLD binding. **c** By associating each Zelda site locus to the nearest TSS, we show that cluster 1 peaks are mostly associated with maternally deposited genes that are also transcribed in early developmental stages (“maternal/zygotic,” e.g., house-keeping genes). Cluster 2 is more associated with zygotically transcribed genes, while cluster 3 is mostly not expressed [30]. **d** Average expression levels [30] for gene associated with clustered Zelda peaks, at eight time points throughout MZT. **e** Different chromatin patterns as annotated into five chromatin types using HMM with 53 chromatin proteins in Drosophila Kc167 cells [21]. Types include tissue-specific active genes (*red*), “house-keeping”-like active genes (*yellow*), and heterochromatin (*green*), polycomb-related (*blue*) or silenced genomic regions (*black*)

First, we wanted to test whether the detected differences in chromatin packaging affect transcription factor binding. For this, we analyzed genome-wide binding data for 17 early development transcription factors, as measured after the completion of the maternal-to-zygotic transition at mitotic cycle 14 [13]. For each of the 2000 early Zelda peaks that were clustered, we calculated the number of unique anterior–posterior (A–P) and dorsal–ventral (D–V) transcription factors that are bound at the same locus. As shown in Fig. 5a, early Zelda-bound regions in cluster 1 show strong accumulation of transcription factors by cycle 14 (>4 TFs on average), with an even stronger signal for cluster 2 Zelda sites (~6 TFs on average), compared to ~2 TFs for cluster 3 regions.

This raises the hypothesis that while most early Zelda sites become accessible during MZT and facilitate the binding of regulatory proteins, perhaps the sites captured by cluster 3 do not.

For this, we turned to examine the dynamics of Zelda binding itself, as measured by ChIP-seq in three time points throughout MZT [16]. As shown in Fig. 5b, a similar number of early Zelda peaks in cluster 1 exhibit increased vs. decreased ChIP signal during MZT (37% increasing, 40% decreasing). Cluster 2 Zelda peaks are mostly increasing (50%, compared to 28% peaks with weaker ZLD ChIP-seq signal from mitotic cycle 8–14). Conversely, the vast majority (79%) of Zelda peaks in cluster 3 show a decrease in Zelda binding, consistently with the observed reduction in transcription factor binding. As shown in Additional file 2: Figure S2, cluster 3 Zelda peaks are initially showing a lower average ChIP-seq signal for Zelda binding, and indeed most peaks show a consistent decrease throughout the MZT.

To test the functional effect of these three groups in regulating gene expression, we associated every Zelda peak with the nearest gene. Indeed, we observed differences in the distance distribution to the nearest TSS. While over 38% of cluster 1 peaks were directly located at gene promoters (hence the asymmetric H3K4me3 signature shown in Fig. 4, left), only 25% of the peaks from cluster 2, and 11% of cluster 3 Zelda peaks were located at promoters (Additional file 3: Figure S3). Opposite trends were observed for distal “intergenic” peaks, consisting of 3% of cluster 1 peaks, compared to 23 and 25% of clusters 2 and 3, respectively. Considering the chromatin signature (H3K4me1 and H3K27me3) and the high number of TF bound at these loci, it is not unlikely that most of the distal regions (in cluster 2) act as regulatory enhancers.

Following Harrison et al. [16], where we used single-embryo RNA-seq data from mitotic cycles 10–14 [30] to classify early developmental genes into temporal groups, we have now compared these annotations to the genes associated with each of the peaks in the three clusters. As shown in Fig. 5c, peaks in cluster 1 were exclusively associated with genes expressed both maternally and in the zygote (maternal/zygotic) or early developmental zygotic genes. Zelda peaks from cluster 2 (i.e., the putative enhancer-like group) were even more enriched with early, strictly zygotic genes. Cluster 3 was dramatically enriched with silenced genes or house-keeping genes (expressed both maternally and in the zygote). As Fig. 5d and Additional file 4: Figure S4A show, genes associated with cluster 1 peaks are already expressed (strongly) by mitotic cycle 10 [30], while cluster 2 Zelda peaks are mostly associated with early developmental genes activated during MZT.

Finally, we compared the three clusters to HMM-based annotations of the fly genome into five chromatin types, based on the binding patterns of 53 chromatin proteins in Drosophila Kc167 cells [21]. While these results are based on a cultured cell-line—compared to in vivo ChIP-seq or gene expression data from embryos—they do originate from embryonic tissue (developmental stages 13–15). These results demonstrate how early Zelda binding shapes the chromatin to form the basis for later stages of embryonic development. As shown in Fig. 5e, the three classes show district patterns, with cluster 1 mostly enriched for the active genes (either “house-keeping”-like genes in yellow, or more tissue-specific genes, red), as well as genomic regions that are packed in polycomb (blue) or repressed (black) in Kc167 cells.

Functional GO annotation analysis for the genes associated with each Zelda cluster also supports our separation into three classes, where cluster 1 peaks are associated with terms such as anatomical morphogenesis and developmental genes (*p* < 8e−28 and *p* < 3.3e−22, respectively), while cluster 2 peaks are more associated with transcription factors (*p* < 5.6e−45) and patterning (*p* < 1.1e−40; Additional file 4: Figure S4).

### De novo chromatin-based identification of Zelda-like loci

We next turned to use the three chromatin signatures obtained using the spectral clustering algorithm and identify additional loci showing similar chromatin patterns. While Zelda plays a major role in shaping the chromatin landscape and accessibility during the MZT, marking them for activation and enabling the binding of regulatory proteins, many early Drosophila genes are still expressed in Zelda maternal mutants embryos [5, 20]. This suggests that there might be some other mechanisms (besides Zelda) that may also lead to similar chromatin signatures of active and regulatory chromatin (Additional file 5: Figure S5, Additional file 6: Figure S6).

For this, we cross-correlated the genome with the chromatin patterns of the promoter-like (cluster 1) and enhancer-like (cluster 2) signatures (Fig. 4). We used the same set of histone modification marks as used for clustering (H3K27ac, H3K27me3, H3K4me1, H3K4me3, and H3K18ac) at cycle 14, with the same 10 Kb windowing. Initially, we scanned the genome with each of the histone modification patterns separately and computed the average correlation for all modifications. This naive analysis failed to obtain satisfying results, as the relative strength of different histone modification (ChIP-seq signal) was not preserved. As a result, many loci showed high correlation all query patterns, but their combined chromatin signature was skewed (e.g., strong H3K27me3 flanks, with very weak H3K4me1 signal). Next, we tried to search for all modifications simultaneously by concatenating the histone modifications into one long vector and then cross-correlating against the genome. These results were still rather disappointing, since correlation by itself does not account for the overall magnitude of the histone modification pattern, but only the overall shape.

As a trade-off between shape and strength consideration, we added a regularization term that combined the correlation coefficient obtained for each genomic locus (Eq. 7) by the empirical likelihood (or the relative frequency) of the height of ChIP-seq signal, among the bona fide Zelda peaks. This reflects the empirical prior probability of obtaining a peak of such height among positive samples.

**Fig. 6:**
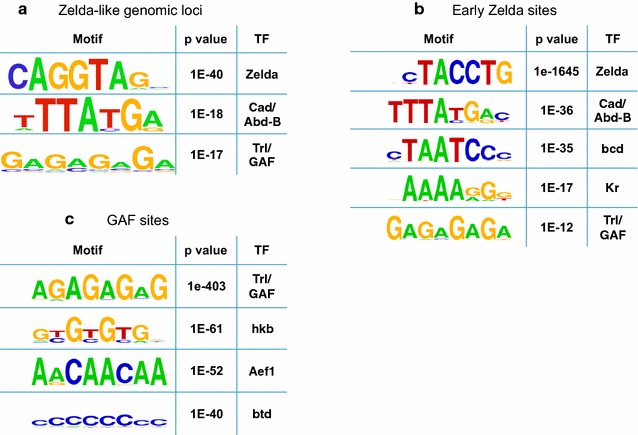
De novo motif analysis for top putative enhancer-like regions. **a** Motif analysis of top 1600 putative enhancer-like regions using HOMER [32]. Using 100 bp windows, identified the CAGGTAG motif (Zelda binding site, *p* < 1e−40), a second motif (*p* < 1e−18, possibly related to Caudal or Abd-B), and poly(GA), bound by GAGA factor (*p* < 1e−17; GAF, Trl). **b** Similar motif analysis for 2000 early Zelda peaks and (C) ~6000 GAF sites

We then scanned the *D. melanogaster* genome at both orientations and extracted genomic loci with high local score. As shown in Fig. 6, our de novo chromatin-based identification of Zelda-like loci retrieved the majority of bona fide Zelda peaks in clusters 1 and 2. When considering the top 2,500 hits identified by the promoter-like (cluster 1) signature, we identified over 75% of the original Zelda peaks in that cluster, with the addition of ~400 (15%) regions with similar chromatin patterns. Similarly, by scanning the genome with the enhancer-like (cluster 2) pattern, we managed to locate more than 80% of original early Zelda peaks, with an addition of ~200 (7%) loci not associated with Zelda peaks. When examining in vivo binding of A–P and D–V transcription factors (as before), we observed an average of 5.2 bound factors among the novel enhancer-like regions (compared to ~6 TFs found at the top Zelda-bound regions and <2 factors bound at random regions).

### Motif analysis of discovered putative regulatory sites

To further examine the functional role of the newly discovered enhancer-like regions, we ran a de novo motif analysis on the top 1600 enhancer-like peaks, using HOMER [32]. Our motif analysis, based on 100 bp long sequences centered on the mid-points of “hit” 10 Kb windows, identifying three major motifs de novo (Fig. 6a). Motif analysis with 250 bp or 500 bp long sequences yielded similar results. The most significant motif identified among Zelda-like regions is CAGGTAG (*p* < 1e−40), the known recognition element of Zelda [14–17]. This enrichment is not surprising, considering the enrichment of Zelda sites within our set of chromatin-based enhancer-like loci. The second motif identified by HOMER [32], with a *p* value of 1e−18, is TTATGA and is similar to the known recognition site of two *Drosophila* proteins, Caudal (Cad) and Abdominal-B (Abd-B). The latter shows very low expression levels, and only toward the end of cycle 14 [30], while the former is a key transcription factor that is expressed in a gradient toward the posterior end of early Drosophila embryos and acts as a morphogen for abdomen/tail formation. Motif 3, with a *p* value of 1e−17, is a poly(GA)-rich motif and is similar to the known recognition site of the protein GAGA factor (GAF, also referred to as Trithorax-like, or Trl).

To determine whether Caudal or GAGA factor (GAF) may act as pioneer factors independently of Zelda, we examined ChIP-seq and ChIP-chip data for Caudal and GAF and calculated their overlap with Zelda peaks [13, 16, 24]. Overall, almost all Caudal peaks (94%) seem to overlap with Zelda binding, compared to only half (56%) of GAF peaks. We therefore continued to study the role of GAF as a pioneer factor.

For comparison, similar motif analysis (Fig. 6b) for the initial set of 2000 early Zelda peaks identified CAGGTAG (*p* < 1e−1645), Caudal (*p* < 1e−36), bicoid (*p* < 1e−35), Kruppel (*p* < 1e−17) and GAF (Trl, *p* < 1e−12, barely above the significant threshold following correction for multiple hypothesis testing). Similar analysis for 5927 GAF binding sites (Fig. 6c) identifies the Trl/GAF motif (*p* value <1e−403), as well as the recognition motifs for hkb (*p* < 1e−61), Aef1 (*p* < 1e−52), btd (*p* < 1e−52).

### GAGA factor (GAF) acts as a Zelda-like pioneer factor

As our motif analysis showed, the GAGA motif (to which GAF binds) is enriched among the putative enhancer-like loci, with about half of GAF sites showing no Zelda binding. To test how prevalent GAF binding is at those enhancer-like loci, we also analyzed GAF ChIP data from hours 0–8 of *D. melanogaster* development [24]. Indeed, the average ChIP strength for the predicted enhancer-like loci is more than threefold higher, compared to flanking regions (77 vs. 22). It should be noted that similarly to the motif analysis where only ~15% of the enhancer-like regions contained a GAGA motif, analysis of ChIP data identified GAF binding in about 20% of these regions (compared to over 75% for Zelda binding).

Finally, we turned to directly test the ability of GAF to act as a pioneer factor with or without Zelda. Recently, we measured DNA accessibility via formaldehyde-assisted isolation of regulatory elements (FAIRE) experiments [20]. The data consist of FAIRE throughout 2–3 h of development in wild-type embryos, as well as embryos maternally depleted for Zelda (zld^M−^).

To test the hypothesis that GAF and Zelda could both act as a pioneer factors, we decided to analyze ZLD and GAF binding to every putative enhancer-like region and to compare these data with their overall DNA accessibility data (as measured by FAIRE) in WT and in zld^M−^ mutants.

For ZLD, we expected to see a positive correlation between ZLD binding and DNA accessibility (i.e., regions with strong ZLD binding are more likely to be accessible) in WT, but not in zld^M−^ mutants. In addition, we expect to see that enhancers with strong GAF binding and weak Zelda binding are less affected by the deletion of *zld*, namely to show strong accessibility in both WT and zld^M−^ mutants FAIRE data.

**Fig. 7:**
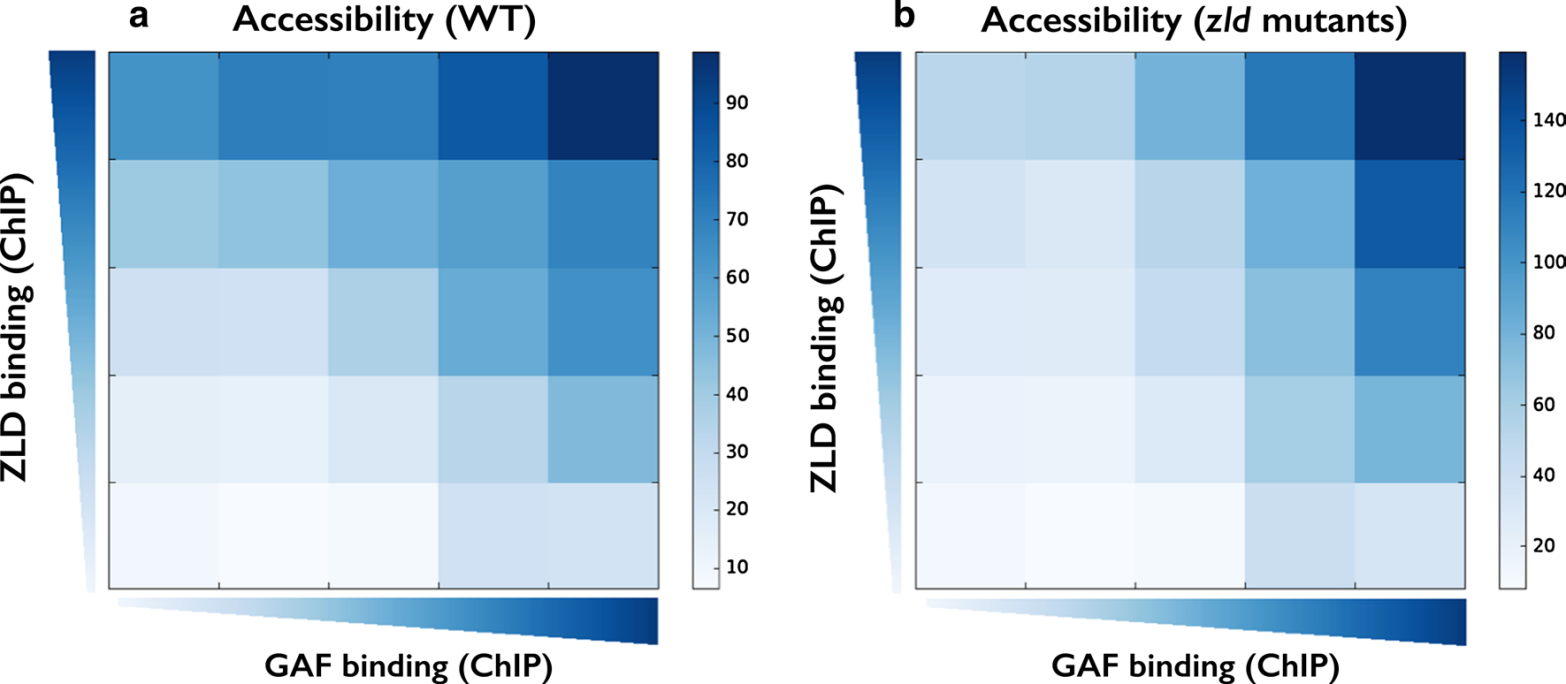
DNA accessibility in WT and zld mutants. **a** Shown is the average FAIRE signal (DNA accessibility) in WT embryos for 2500 enhancer-like loci that are binned based on their ZLD (*Y*-axis) and GAF (*X*-axis) ChIP binding. **b** Same as A, using FAIRE data from maternal zld mutants [20]. Comparison of the two matrices shows that genomic regions with no GAF binding (in WT) are mostly affected by deletion of zld (left hand side of both matrices), while regions bound by GAF (right hand side) still show high DNA accessibility, suggesting that GAF could ac as a pioneer factor in early fly development, together or independently of Zelda

Due to the relatively low number of data points (2500 enhancer-like regions divided into 36 groups according to their ZLD and GAF ChIP signals), we only show the average FAIRE accessibility for each group. As Fig. 7a shows for the WT embryos, an increase in both ZLD binding (moving upwards) and GAF binding (moving to the right), results in increased DNA accessibility (darker shade of blue). Conversely, the accessibility matrix for zld^M−^ mutants is less sensitive to ZLD levels, especially on the left hand side, where GAF levels are very low, yet it is almost unaffected by the deletion of zld among the strong GAF sites (Fig. 7b, right hand side). These data suggest that GAGA factor may act as a pioneer factor and contribute to the accessibility and chromatin landscape of DNA regions, much like Zelda (yet for fewer genomic loci), independently of Zelda.

## Discussion

In this work, we developed a computational method aimed to analyze the chromatin landscape surrounding some regions of interest (here, binding of the protein Zelda in early embryonic development of the fruit fly *D. melanogaster*), refined it by clustering into sub-groups of unique characteristics, and then scanned the entire genome for novel regions showing similar chromatin characteristics.

As we have shown, much of our method’s accuracy stems from the initial re-orientation and clustering procedure, which together allow us to derive a specialized mixture model rather than averaging away the chromatin signal.

We have devised methodologies to simultaneously analyze multiple genomic tracks (i.e., different histones modifications) at different time points, while maintaining the spatial signature of each modification. Such models are more compelling than naive combinatorial models that only consider the heights of the various modifications but ignore the overall shape. As we have shown, many of the observed signals come in unique shapes, including a dip at the ZLD site with two flanking nucleosomes marked with one modification, all within some domain of different modification.

We have analyzed early Zelda peaks throughout the Drosophila genome and identified three typical chromatin signatures they are associated with, including (1) active promoters, (2) active enhancer regions, and (3) genomic regions that were transiently bound by Zelda. We then characterized each of the classes and scanned the genome for additional regions matching the enhancer-like chromatin signature. As we have shown, many of the retrieved regions were shown to be bound by either Zelda or GAF, suggesting both act as pioneer factors by making these genomic regions accessible and depositing the appropriate histone modifications, thus marking them for activation during the maternal-to-zygotic transition. The entire role of GAF in MZT as well as the possible role for additional factors is yet to be studied.

While these questions are key to understanding the processes that initiate and shape embryonic gene expression in the fruit fly *D. melanogaster*, the method presented here is more general and would allow—given a query set of input loci—the identification of similar elements in any genome. In that sense, we believe that our method could be of great interest to any genome-wide research project.

## Conclusions

As more genome-wide chromatin data accumulate in multiple conditions, computational methods emerge as a possible means to characterize key loci and identify novel genomic regions with similar chromatin signatures. Our work addresses these tasks by re-orienting and clustering the chromatin landscape surrounding regions of interest and then scanning the entire genome for novel regions showing similar characteristics. Using pre-MZT regions bound by the pioneer factor Zelda, we identify thousands of additional Zelda-like enhancer regions and mark the protein GAF (Vfl) as an additional factor in regulation of early developmental genes. These results are significant as they untangle the complexity in chromatin signatures near Zelda sites, and in linking GAF to chromatin accessibility and gene regulation, as well as developing a novel end-to-end method for studying genomic data.

## Methods

### Multichannel chromatin representation of ZLD sites

To characterize the chromatin signature around Zelda sites, we considered the top 2000 Zelda peaks in mitotic cycle 8 [16] and looked at five histone modifications (H3K27ac, H3K27me3, H3K4me1, H3K4me3, and H3K18ac) at two time points (mitotic cycles 13 and 14) [5]. For each peak, we considered a window of 10 Kb (at 10 bp resolution) and constructed a vector of length 1000 for each modification. This resulted in a 10 × 1000 matrix for each Zelda site.

### Chromatin-based re-orientation of Zelda peaks

These 2000 Zelda peaks (namely a 3D matrix of 2000 × 10 × 1000) was subjected to an Expectation Maximization (EM)-like iterative orientation algorithm [26, 29]. For every peak, we randomly sampled an orientation (+/−) and calculate the average ChIP-seq signal. Then, for 20 iterations (or until convergence), we assigned each peak with the orientation minimizing the overall distance to the average chromatin signature (as in the Max–Max variation of the algorithm).

### Peak annotation and GO term analysis

Peaks were associated with nearest genes and annotated with HOMER [32], using “annotatePeaks.pl” with default parameters (dm3). GO term annotations are based on “annotatePeaks.pl” with the “-go” option.

### Chromatin-based distance and adjacency functions

To define a distance function between two loci ***x***_***i***_ and ***x***_***j***_, we begin by focusing on one histone modification ***m***:

1 $${\text{dist}}_{i,j}^{m} = \sqrt {\frac{{\sum {\left( {\vec{x}_{i}^{m} - \vec{x}_{j}^{m} } \right)^{2} } }}{n}}$$

We then use a standard Gaussian kernel to translate distances into weights in the spectral clustering adjacency matrix

2 $$w_{i,j}^{m} = e^{{ - ({\text{dist}}_{i,j}^{m} )^{2} /2\sigma_{m}^{2} }}$$

where ***σ***_***m***_ is a modification-specific parameter (set below). We then sum the adjacency values over all 10 modifications to obtain one value:

3 $$w_{i,j} = \sum\limits_{{m \in {\text{mods}}}} {w_{i,j}^{m} }$$

### Spectral clustering

Given *N* < 10 × 1000 > matrices **{x**_**i**_ **}** that correspond to the histone modification data for each peak, we defined the graph Laplacian:

4 $$L = D - W$$

where *W* is the adjacency matrix (as defined above, Eq. 4), and *D* is the diagonal degree matrix, defined as:

5 $$D_{i,j} = \left\{ {\begin{array}{*{20}l} 0 &\quad {i \ne j} \\ {\sum\nolimits_{k} {w_{i,k} } } &\quad {i = j} \\ \end{array} } \right.$$

Following Ng, Jordan, and Weiss [31], we transform *L* into the symmetric normalized Laplacian

6 $$L^{\text{sym}} = D^{ - 1/2} LD^{ - 1/2}$$

thus normalizing the rows and columns of the graph Laplacian. We then select the first ***k*** eigenvectors of ***L*** ^***sym***^, project the *N* data points onto this ***k***-dimensional subspace, and normalize the projected data (rows of length ***k***) to *L* ^2^ norm of 1 (i.e., project each data point to the ***k***-dimensional sphere). Finally, we apply a standard k-means clustering algorithm (where ***k*** here equals the number of eigenvectors ***k*** used for the spectral projection).

To choose ***k***, we applied a standard eigengap (or spectral gap) heuristic, namely calculating the difference between successive eigenvalues (**λ**_**k**_ and **λ**_**k+1**_) and choosing the first to surpass some threshold.

### Empirically normalized distance/adjacency function

In Eq. 2 we described the Gaussian kernel used to translate average Euclidean distances into adjacency weights. As each genomic track (histone modification, time) presents different absolute values, it is crucial to normalize each Gaussian specifically using its own scaling parameter *σ*_*m*_. For this, we calculated the empirical distribution of (unnormalized) pairwise distances for each modification m (Additional file 7: Figure S7, purple line), and set *σ*_*m*_ to be the 10th percentile (red vertical line, Additional file 7: Figure S7). This way, we ensured that for each modification exactly 10% of the pairwise distances (purple regions) will have adjacency values larger than exp(−½) = 0.606 in the adjacency matrix. This independently normalizes each modification to its own distribution of pairwise distances, thus setting similar “weight” to each modification, allowing for the summation of different adjacency matrices into one (Eq. 3).

### Genome-wide scanning of similar chromatin loci

Following the spectral clustering of Zelda peak into three classes, we wished to identify novel enhancer regions with similar chromatin signature to that of Zelda cluster 2 peaks. For this, we scanned the genome in 10 Kb windows (stride of 100 bp) and cross-correlated the chromatin signature at each window to the average histone modification pattern of cluster 2 (enhancer-like regions). We scored each locus i by first calculating the Pearson correlation between its surrounding *x*_*i*_^*m*^ (where m denotes the histone modification/time point) versus the chromatin signature for cluster 2 peaks (denoted by cl_2_^*m*^).

This approach, as opposed to just comparing the maximal peak height within each window, gives spatial resolution and identifies the unique shapes we observe for the different modifications (e.g., two symmetric narrow peaks, flanking domains, asymmetrical peak).

To incorporate prior knowledge and capture the overall complex shape of enhancer-like peaks, namely the typical combination of heights for each modification/time point, we also included a “prior” term ***P***:

7 $${\text{Score}}_{i}^{m} = {\text{Corr}}({\text{cl}}_{2}^{m} ,x_{i}^{m} ) \cdot P_{m} ({\text{height}}_{i}^{m} )$$

where **height**_**i**_^**m**^ denotes the average ChIP-seq signal modification **m** within its 10 Kb surrounding locus ***i***, and **P**_**m**_ **(height**_**i**_^**m**^ **)** denotes the empirical probability function (or relative frequency) of such mean ChIP-seq signal for modification ***m*** among bona fide Zelda peaks, as calculated using the chromatin signatures around the initial set of 2000 early Zelda peaks within cluster 2. This penalizes loci with similar shape (hence, high Pearson correlation coefficients) but in different ChIP magnitude.

Finally, we calculated the overall similarity score for each locus, by summing over the modification-specific scores:

8 $${\text{Score}}_{i} = \sum\limits_{m} {{\text{Score}}_{i}^{m} }$$

and identified the top local 2500 maxima genome wide.

## Additional files

**Additional file 1: Figure S1.** Chromatin signatures around clustered early Zelda peaks. Heatmap and average ChIP-seq signal for five histone modifications at two time points (mitotic cycle 13, left) and 14 (right) around top 2,000 early Zelda peaks. Peaks were divided into three clusters, including cluster 1 (blue line; top heatmaps), cluster 2 (orange line; center heatmaps), and cluster 3 (yellow line, bottom heatmaps).

**Additional file 2: Figure S2.** Average ChIP-seq binding of Zelda. (A) Average ZLD ChIP-seq signal at three time points, including mitotic cycle 8 (1 h after fertilization), mitotic cycle 11 (2 h), and mitotic cycle 14 (3 h) for clustered Zelda peaks. (B) Same, shown as metaplot (top) or heatmaps (top heatmap: cluster 1; middle: cluster 2; bottom: cluster 3).

**Additional file 3: Figure S3.** Functional annotation of Zelda peaks. Early 2,000 Zelda peaks (in three clusters) were annotated using HOMER into several classes including promoter/TSS, Exonic, Intronic, and Intergenic loci.

**Additional file 4: Figure S4.** Gene expression and GO term annotations. (A) Transcription levels of gene associated with early 2,000 Zelda peaks (in three clusters), along eight time points throughout the maternal-to-zygotic transition from mitotic cycle 10–14D [30]. (B–D) GO term enrichments for gene associated with cluster 1 Zelda peaks (B); cluster 2 Zelda peaks (C); and cluster 3 Zelda peaks (D).

**Additional file 5: Figure S5.** Analysis of chromHMM states. Chromatin data were analyzed by chromHMM [33] by first binarizing the chromatin data (default parameters) and then segmenting the genome into seven chromatin classes. (A) Shown are the average number of A–P and D–V transcription factors bound for each state. (B) chromHMM regions were associated with genes, and the average expression levels along MZT is shown as in Fig. 5d.

**Additional file 6: Figure S6.** Chromatin signatures and functional annotations of GAF peaks. 5,927 GAF peaks from in vivo GAF binding in *Drosophila melanogaster* embryos (hours 0–8 of development) [24] were analyzed, similarly to our analysis of 2,000 early Zelda peaks. (A) Peaks were re-oriented and clustered into three clusters. Also shown are ZLD in vivo binding data, similarly to Additional file 2: Figure S2. (B) Annotation of GAF peaks, in clusters, shows enrichment of promoters/TSS (41–60% of peaks), and intronic (22–35%) peaks.

**Additional file 7: Figure S7.** Distance–weight functions for various histone marks. Plotted are the empirical pairwise distance distribution (purple line) of various histone modifications at mitotic cycles 13 (bottom) and 14 (top), over pairs of early Zelda peaks <x_i_, x_j_>. Vertical red lines correspond to the 10th percentile in each distance distribution. This value is assigned as σ_m_ (horizontal red line). The blue line shows the matching Gaussian kernel function (based on each σ_m_), used to transform pairwise distances (X-axis) to weights (Y-axis) when building each Laplacian matrix of spectral clustering.

## Abbreviation

MZT: maternal-to-zygotic transition

## References

[1] Kouzarides T (2007). Chromatin modifications and their function. Cell.

[2] Liu CL, Kaplan T, Kim M, Buratowski S, Schreiber SL, Friedman N, Rando OJ (2005). Single-nucleosome mapping of histone modifications in S. cerevisiae. PLoS Biol.

[3] Rada-Iglesias A, Bajpai R, Swigut T, Brugmann SA, Flynn RA, Wysocka J (2011). A unique chromatin signature uncovers early developmental enhancers in humans. Nature.

[4] Heintzman ND, Stuart RK, Hon G, Fu Y, Ching CW, Hawkins RD, Barrera LO, Van Calcar S, Qu C, Ching KA (2007). Distinct and predictive chromatin signatures of transcriptional promoters and enhancers in the human genome. Nat Genet.

[5] Li X-Y, Harrison MM, Villalta JE, Kaplan T, Eisen MB (2014). Establishment of regions of genomic activity during the Drosophila maternal to zygotic transition. eLife.

[6] Kaplan T, Li X-Y, Sabo PJ, Thomas S, Stamatoyannopoulos JA, Biggin MD, Eisen MB (2011). Quantitative models of the mechanisms that control genome-wide patterns of transcription factor binding during early Drosophila development. PLoS Genet.

[7] Thomas S, Li X-Y, Sabo PJ, Sandstrom R, Thurman RE, Canfield TK, Giste E, Fisher W, Hammonds A, Celniker SE (2011). Dynamic reprogramming of chromatin accessibility during Drosophila embryo development. Genome Biol.

[8] Schübeler D (2007). Enhancing genome annotation with chromatin. Nat Genet.

[9] Zaret KS, Carroll JS (2011). Pioneer transcription factors: establishing competence for gene expression. Genes Dev.

[10] Smallwood A, Ren B (2013). Genome organization and long-range regulation of gene expression by enhancers. Curr Opin Cell Biol.

[11] Tadros W, Lipshitz HD (2009). The maternal-to-zygotic transition: a play in two acts. Development.

[12] Li X-Y, Macarthur S, Bourgon R, Nix D, Pollard DA, Iyer VN, Hechmer A, Simirenko L, Stapleton M, Luengo Hendriks CL (2008). Transcription factors bind thousands of active and inactive regions in the Drosophila blastoderm. PLoS Biol.

[13] Macarthur S, Li X-Y, Li J, Brown JB, Chu HC, Zeng L, Grondona BP, Hechmer A, Simirenko L, Keränen SVE (2009). Developmental roles of 21 Drosophila transcription factors are determined by quantitative differences in binding to an overlapping set of thousands of genomic regions. Genome Biol.

[14] ten Bosch JR, Benavides JA, Cline TW (2006). The TAGteam DNA motif controls the timing of Drosophila pre-blastoderm transcription. Development.

[15] Bradley RK, Li X-Y, Trapnell C, Davidson S, Pachter L, Chu HC, Tonkin LA, Biggin MD, Eisen MB (2010). Binding site turnover produces pervasive quantitative changes in transcription factor binding between closely related Drosophila species. PLoS Biol.

[16] Harrison MM, Li X-Y, Kaplan T, Botchan MR, Eisen MB (2011). Zelda binding in the early *Drosophila melanogaster* embryo marks regions subsequently activated at the maternal-to-zygotic transition. PLoS Genet.

[17] Liang H-L, Nien C-Y, Liu H-Y, Metzstein MM, Kirov N, Rushlow C (2008). The zinc-finger protein Zelda is a key activator of the early zygotic genome in Drosophila. Nature.

[18] Nien C-Y, Liang H-L, Butcher S, Sun Y, Fu S, Gocha T, Kirov N, Manak JR, Rushlow C (2011). Temporal coordination of gene networks by Zelda in the early Drosophila embryo. PLoS Genet.

[19] Satija R, Bradley RK (2012). The TAGteam motif facilitates binding of 21 sequence-specific transcription factors in the Drosophila embryo. Genome Res.

[20] Schulz KN, Bondra ER, Moshe A, Villalta JE, Lieb JD, Kaplan T, McKay DJ, Harrison MM (2015). Zelda is differentially required for chromatin accessibility, transcription factor binding, and gene expression in the early Drosophila embryo. Genome Res.

[21] Filion GJ, van Bemmel JG, Braunschweig U, Talhout W, Kind J, Ward LD, Brugman W, de Castro IJ, Kerkhoven RM, Bussemaker HJ (2010). Systematic protein location mapping reveals five principal chromatin types in Drosophila cells. Cell.

[22] Roy S, Ernst J, Kharchenko PV, Kheradpour P, Nègre N, Eaton ML, Landolin JM, Bristow CA, Ma L, modENCODE Consortium (2010). Identification of functional elements and regulatory circuits by Drosophila modENCODE. Science.

[23] Kharchenko PV, Alekseyenko AA, Schwartz YB, Minoda A, Riddle NC, Ernst J, Sabo PJ, Larschan E, Gorchakov AA, Gu T (2011). Comprehensive analysis of the chromatin landscape in *Drosophila melanogaster*. Nature.

[24] Nègre N, Brown CD, Ma L, Bristow CA, Miller SW, Wagner U, Kheradpour P, Eaton ML, Loriaux P, Sealfon R (2011). A cis-regulatory map of the Drosophila genome. Nature.

[25] Sun Y, Nien C-Y, Chen K, Liu H-Y, Johnston J, Zeitlinger J, Rushlow C (2015). Zelda overcomes the high intrinsic nucleosome barrier at enhancers during Drosophila zygotic genome activation. Genome Res.

[26] Kundaje A, Kyriazopoulou-Panagiotopoulou S, Libbrecht M, Smith CL, Raha D, Winters EE, Johnson SM, Snyder M, Batzoglou S, Sidow A (2012). Ubiquitous heterogeneity and asymmetry of the chromatin environment at regulatory elements. Genome Res.

[27] Wang J, Lunyak VV, Jordan IK (2012). Chromatin signature discovery via histone modification profile alignments. Nucleic Acids Res.

[28] Rajagopal N, Xie W, Li Y, Wagner U, Wang W, Stamatoyannopoulos J, Ernst J, Kellis M, Ren B (2013). RFECS: a random-forest based algorithm for enhancer identification from chromatin state. PLoS Comput Biol.

[29] Dempster A, Laird N, Rubin D (1977). Maximum likelihood from incomplete data via the EM algorithm. J Roy Stat Soc Ser B (Methodol).

[30] Lott SE, Villalta JE, Schroth GP, Luo S, Tonkin LA, Eisen MB (2011). Noncanonical compensation of zygotic X transcription in early *Drosophila melanogaster* development revealed through single-embryo RNA-seq. PLoS Biol.

[31] Ng AY, Jordan MI, Weiss Y (2001). On spectral clustering: analysis and an algorithm. NIPS.

[32] Heinz S, Benner C, Spann N, Bertolino E, Lin YC, Laslo P, Cheng JX, Murre C, Singh H, Glass CK (2010). Simple combinations of lineage-determining transcription factors prime cis-regulatory elements required for macrophage and B cell identities. Mol Cell.

[33] Ernst J, Kellis M (2012). ChromHMM: automating chromatin-state discovery and characterization. Nat Methods.

