# Supplementary figures and images for: Genome-wide search for Zelda-like chromatin signatures identifies GAF as a pioneer factor in early fly development
Source: Epigenetics Chromatin. 2017 Jul 4;10:33. doi: 10.1186/s13072-017-0141-5 (PMC5496641; doi:10.1186/s13072-017-0141-5)

# Supplemental Figure 1

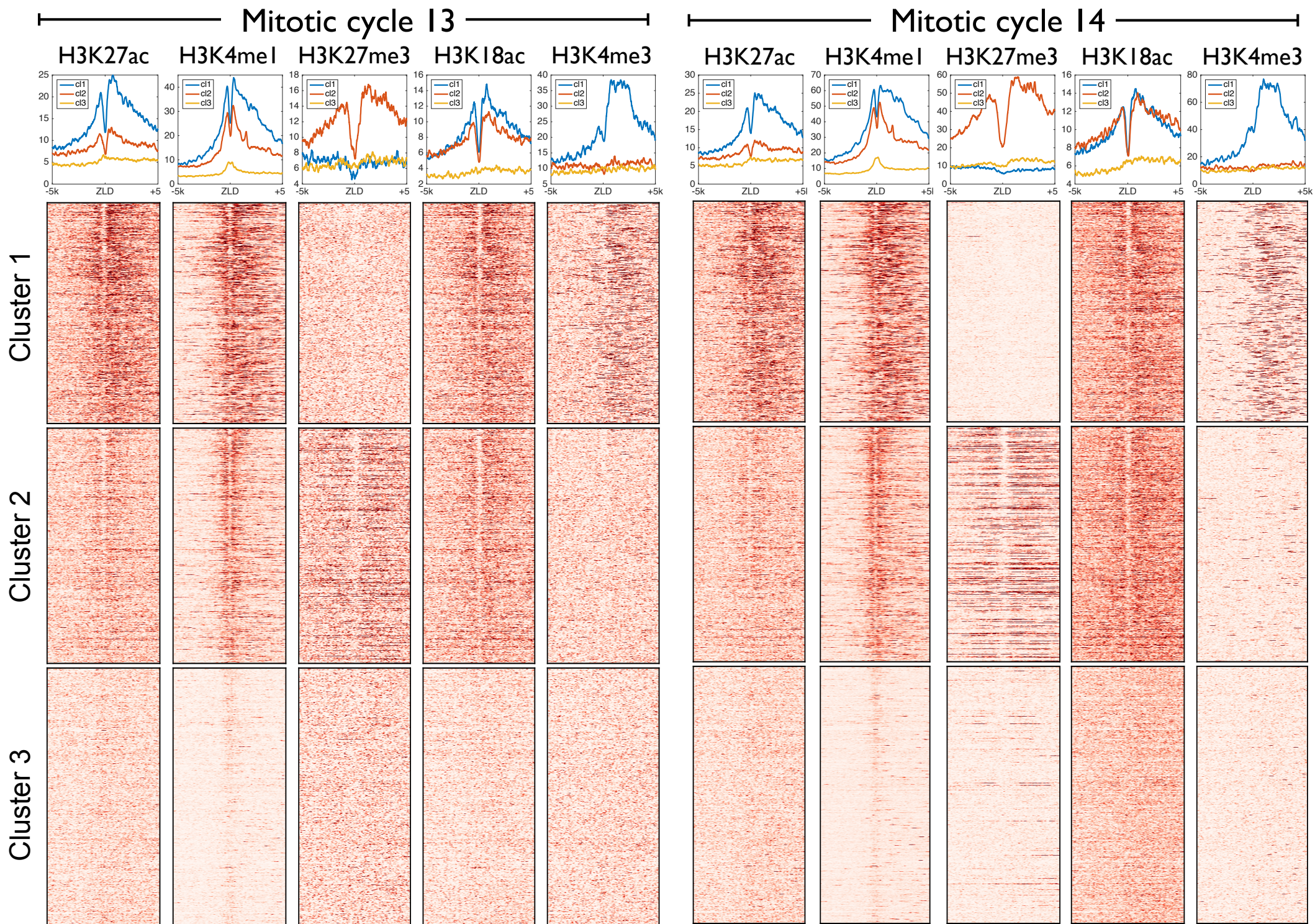

Supplement: Supplementary file 1 — Additional file 1: Figure S1. Chromatin signatures around clustered early Zelda peaks. Heatmap and average ChIP-seq signal for five histone modifications at two time points (mitotic cycle 13, left) and 14 (right) around top 2,000 early Zelda peaks. Peaks were divided into three clusters, including cluster 1 (blue line; top heatmaps), cluster 2 (orange line; center heatmaps), and cluster 3 (yellow line, bottom heatmaps). [file 13072_2017_141_MOESM1_ESM.pdf]

# Supplemental Figure 2

(A)

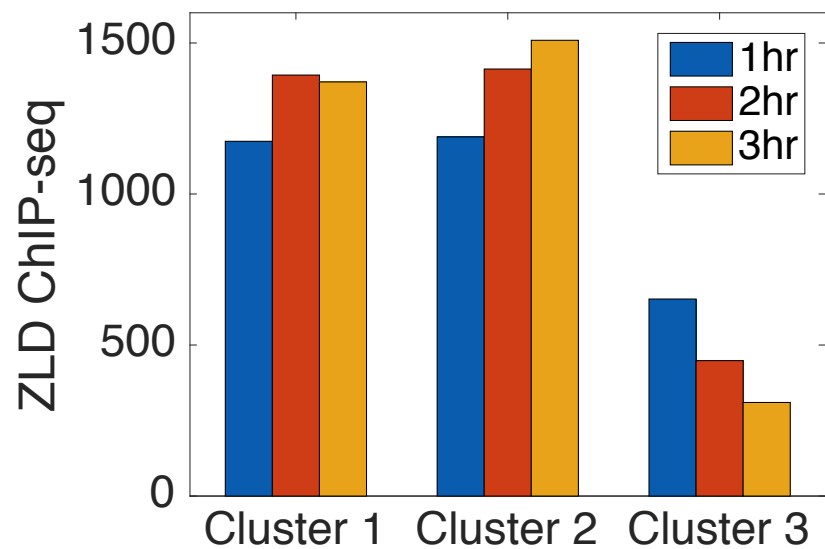

(B)

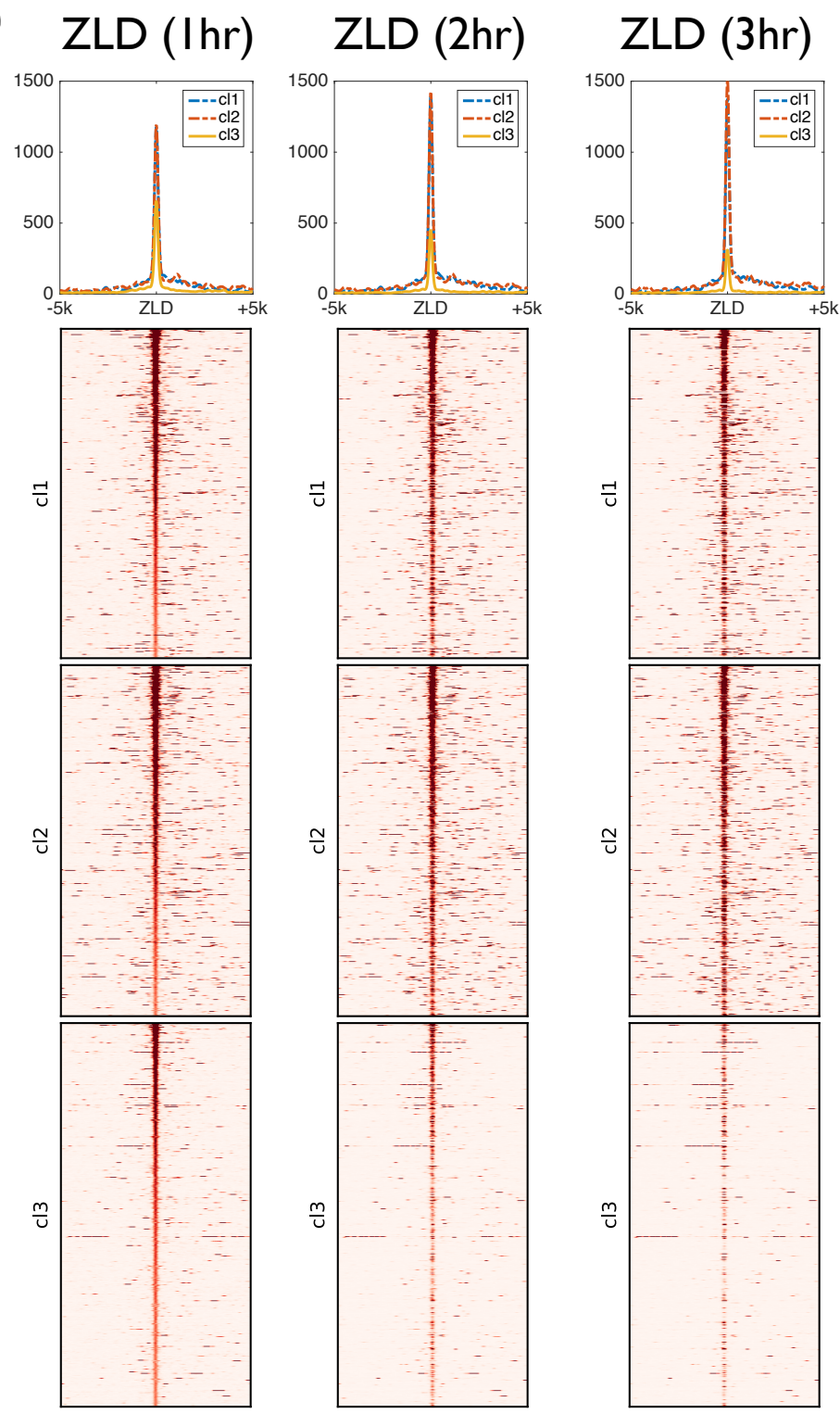

Supplement: Supplementary file 2 — Additional file 2: Figure S2. Average ChIP-seq binding of Zelda. (A) Average ZLD ChIP-seq signal at three time points, including mitotic cycle 8 (1 h after fertilization), mitotic cycle 11 (2 h), and mitotic cycle 14 (3 h) for clustered Zelda peaks. (B) Same, shown as metaplot (top) or heatmaps (top heatmap: cluster 1; middle: cluster 2; bottom: cluster 3). [file 13072_2017_141_MOESM2_ESM.pdf]

# Supplemental Figure 3

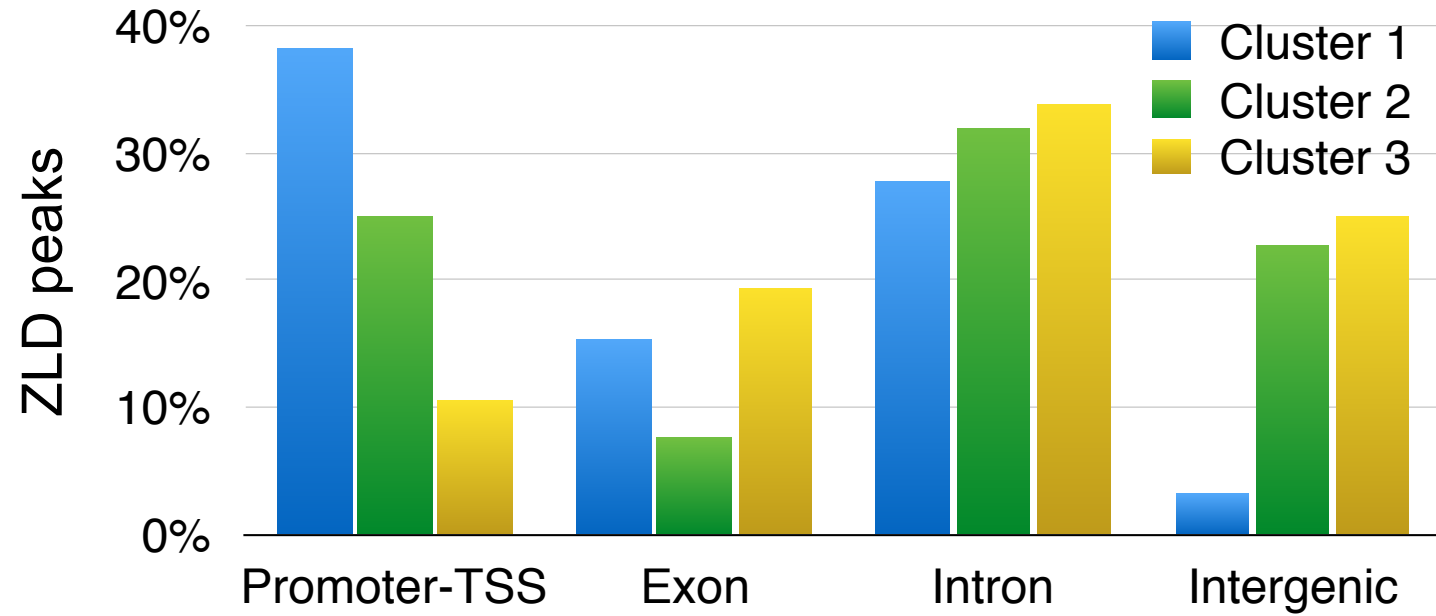

Supplement: Supplementary file 3 — Additional file 3: Figure S3. Functional annotation of Zelda peaks. Early 2,000 Zelda peaks (in three clusters) were annotated using HOMER into several classes including promoter/TSS, Exonic, Intronic, and Intergenic loci. [file 13072_2017_141_MOESM3_ESM.pdf]

# Supplemental Figure 5

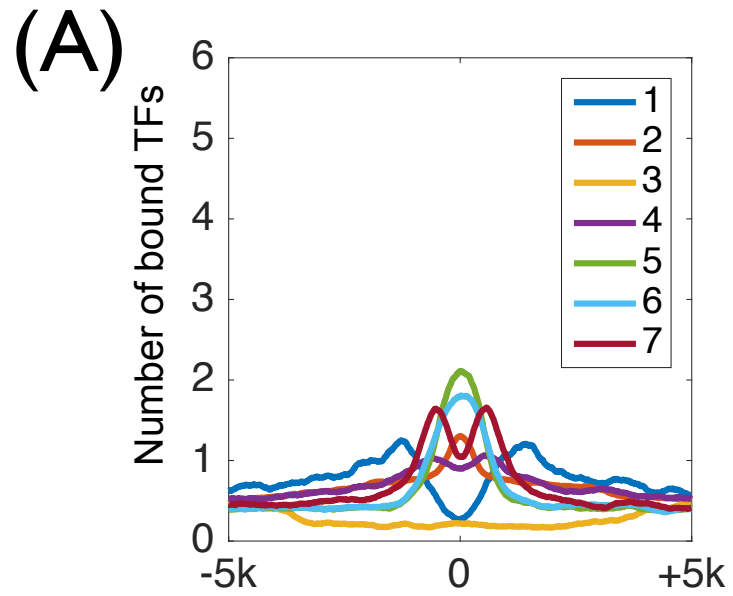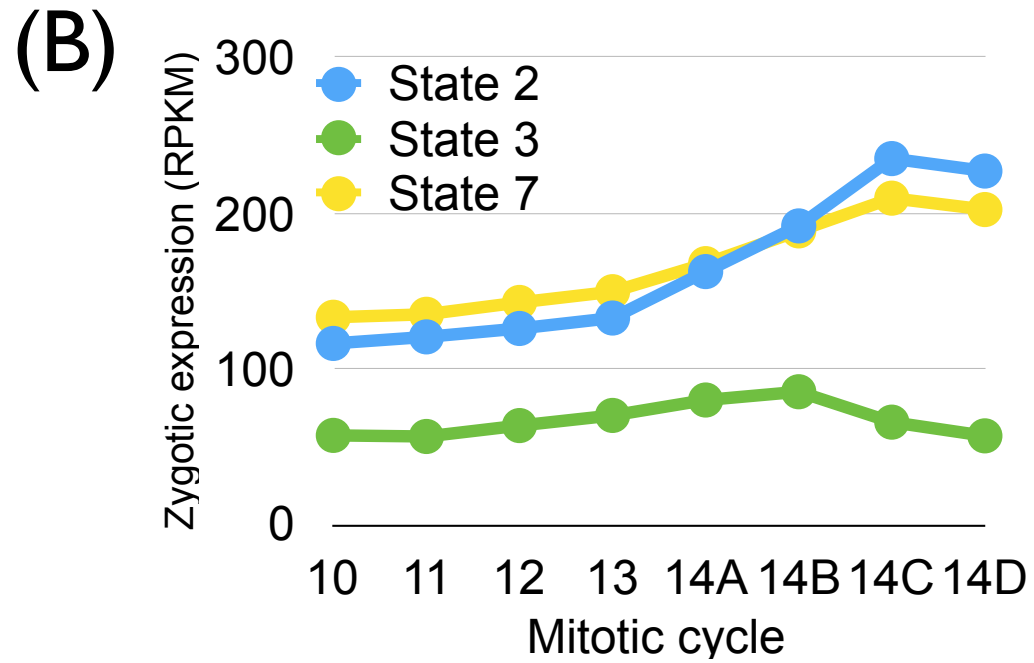

Supplement: Supplementary file 5 — Additional file 5: Figure S5. Analysis of chromHMM states. Chromatin data were analyzed by chromHMM [33] by first binarizing the chromatin data (default parameters) and then segmenting the genome into seven chromatin classes. (A) Shown are the average number of A–P and D–V transcription factors bound for each state. (B) chromHMM regions were associated with genes, and the average expression levels along MZT is shown as in Fig. 5d. [file 13072_2017_141_MOESM5_ESM.pdf]

# Supplemental Figure 6

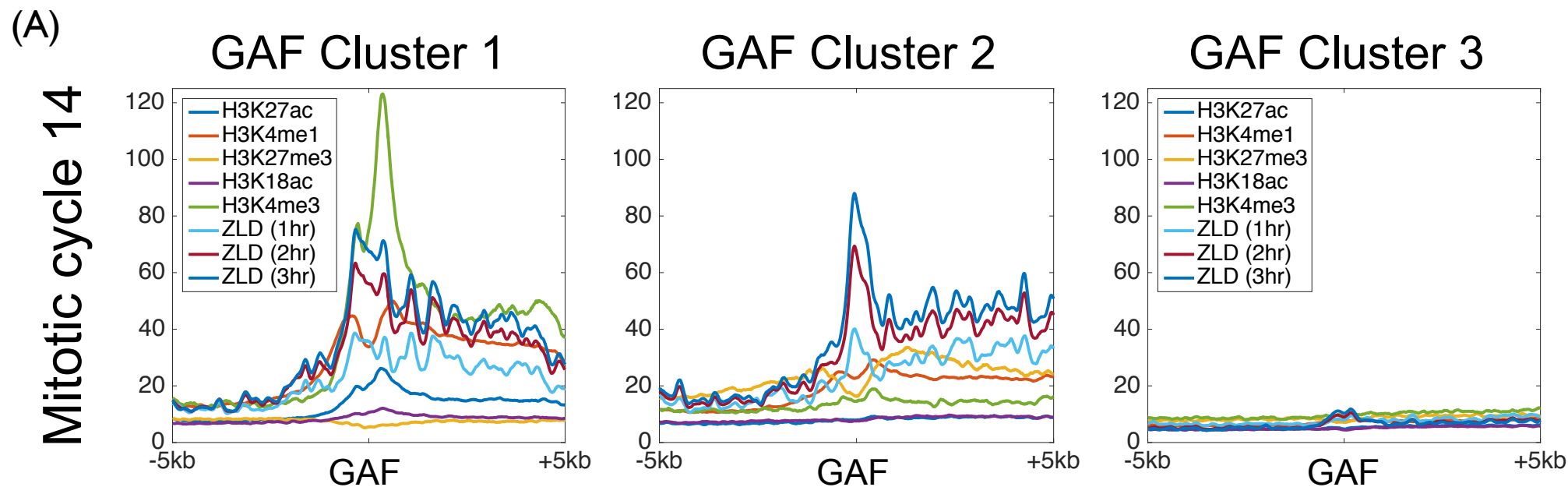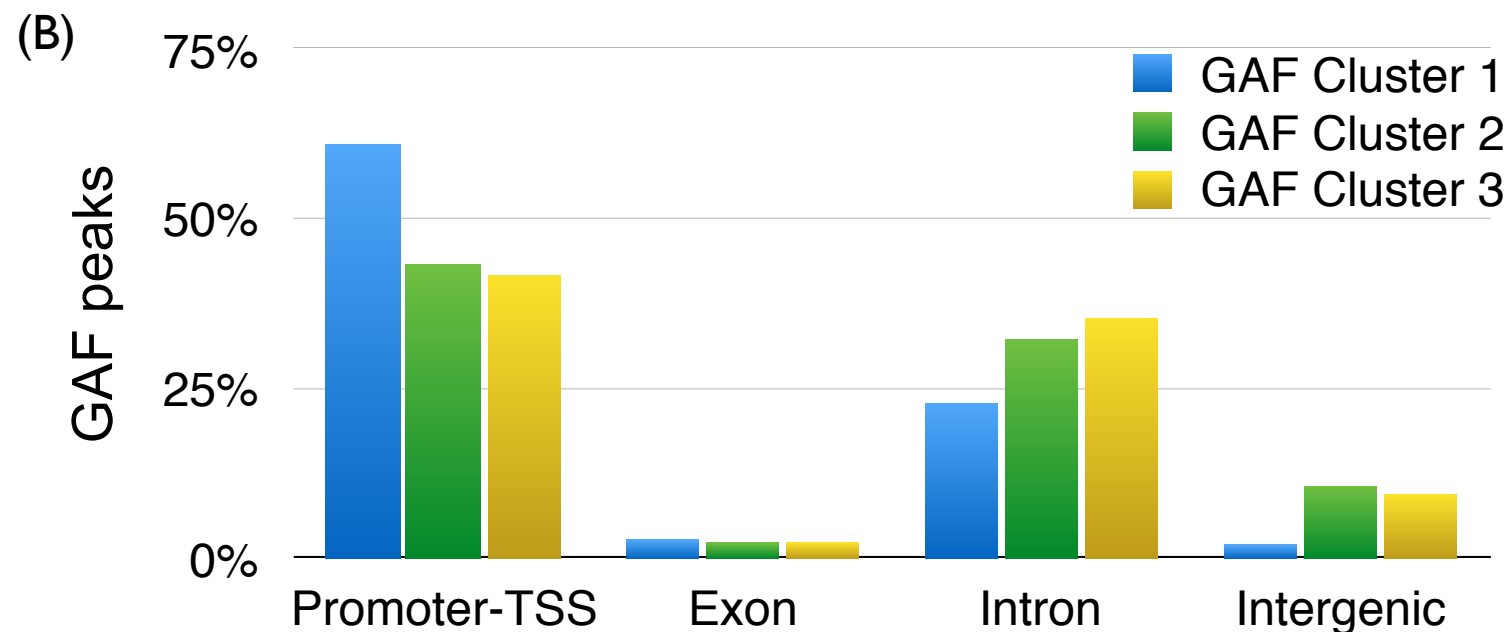

Supplement: Supplementary file 6 — Additional file 6: Figure S6. Chromatin signatures and functional annotations of GAF peaks. 5,927 GAF peaks from in vivo GAF binding in Drosophila melanogaster embryos (hours 0–8 of development) [24] were analyzed, similarly to our analysis of 2,000 early Zelda peaks. (A) Peaks were re-oriented and clustered into three clusters. Also shown are ZLD in vivo binding data, similarly to Additional file 2: Figure S2. (B) Annotation of GAF peaks, in clusters, shows enrichment of promoters/TSS (41–60% of peaks), and intronic (22–35%) peaks. [file 13072_2017_141_MOESM6_ESM.pdf]

# Supplemental Figure 7

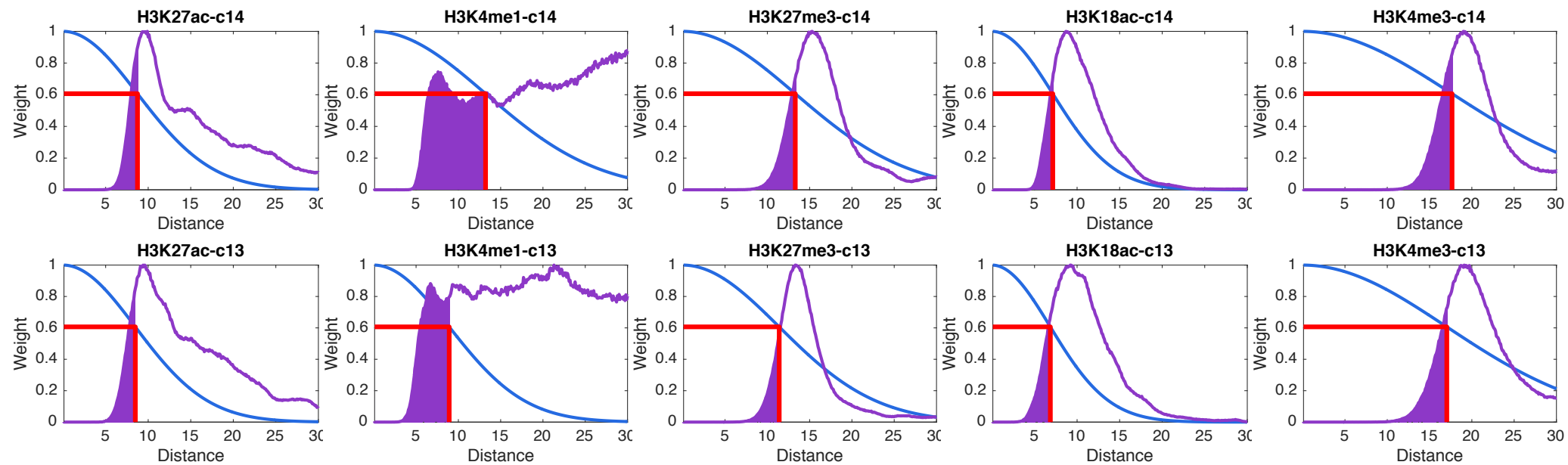

Supplement: Supplementary file 7 — Additional file 7: Figure S7. Distance–weight functions for various histone marks. Plotted are the empirical pairwise distance distribution (purple line) of various histone modifications at mitotic cycles 13 (bottom) and 14 (top), over pairs of early Zelda peaks <xi, xj>. Vertical red lines correspond to the 10th percentile in each distance distribution. This value is assigned as σm (horizontal red line). The blue line shows the matching Gaussian kernel function (based on each σm), used to transform pairwise distances (X-axis) to weights (Y-axis) when building each Laplacian matrix of spectral clustering. [file 13072_2017_141_MOESM7_ESM.pdf]
