# Supplementary material for: Genome-wide search for Zelda-like chromatin signatures identifies GAF as a pioneer factor in early fly development
Source: Epigenetics Chromatin. 2017 Jul 4;10:33. doi: 10.1186/s13072-017-0141-5 (PMC5496641; doi:10.1186/s13072-017-0141-5)
Supplement: Supplementary file 4 — Additional file 4: Figure S4. Gene expression and GO term annotations. (A) Transcription levels of gene associated with early 2,000 Zelda peaks (in three clusters), along eight time points throughout the maternal-to-zygotic transition from mitotic cycle 10–14D [30]. (B–D) GO term enrichments for gene associated with cluster 1 Zelda peaks (B); cluster 2 Zelda peaks (C); and cluster 3 Zelda peaks (D). [file 13072_2017_141_MOESM4_ESM.pdf]

Supplemental Figure 4

(A)

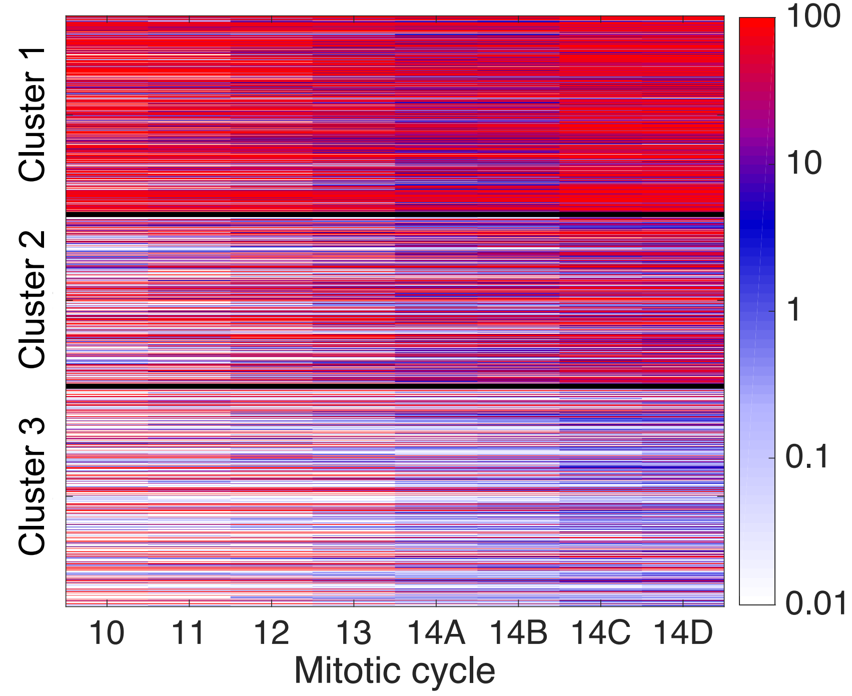

(B)

Cluster 1

GO Term	p-value
anatomical structure morphogenesis	8E-28
anatomical structure development	3.3E-22
organ development	4E-22
system development	3E-20
developmental process	5.3E-20

(C)

Cluster 2

GO Term	p-value
sequence-specific DNA binding transcription factor activity	5.6E-45
pattern specification process	1.1E-40
regulation of transcription	3.5E-40
regionalization	3.8E-40
organ development	1.3E-38

(D)

Cluster 3

GO Term	p-value
cell morphogenesis involved in differentiation	1.7E-08
cell morphogenesis involved in neuron differentiation	9E-07
nucleic acid binding transcription factor activity	6.6E-06
